# A Scoping Review of Technological Approaches to Environmental Monitoring

**DOI:** 10.3390/ijerph17113995

**Published:** 2020-06-04

**Authors:** Graham Coulby, Adrian Clear, Oliver Jones, Alan Godfrey

**Affiliations:** 1 Department of Computer and Information Sciences, Faculty of Engineering and Environment, Northumbria University, Newcastle Upon Tyne NE1 8ST, UK; g.d.coulby@northumbria.ac.uk (G.C.); adrian.clear@northumbria.ac.uk (A.C.); 2 Department of Technologies, Ryder Architecture, Newcastle Upon Tyne NE1 3NN, UK; ojones@ryderarchitecture.com

**Keywords:** commercial building, residential building, Internet of Things (IoT), health, wellbeing, indoor environment quality (IEQ)

## Abstract

Indoor environment quality (IEQ) can negatively affect occupant health and wellbeing. Air quality, as well as thermal, visual and auditory conditions, can determine how comfortable occupants feel within buildings. Some can be measured objectively, but many are assessed by interpreting qualitative responses. Continuous monitoring by passive sensors may be useful to identify links between environmental and physiological changes. Few studies localise measurements to an occupant level perhaps due to many environmental monitoring solutions being large and expensive. Traditional models for occupant comfort analysis often exacerbate this by not differentiating between individual building occupants. This scoping review aims to understand IEQ and explore approaches as to how it is measured with various sensing technologies, identifying trends for monitoring occupant health and wellbeing. Twenty-seven studies were reviewed, and more than 60 state-of-the-art and low-cost IEQ sensors identified. Studies were found to focus on the home or workplace, but not both. This review also found how wearable technology could be used to augment IEQ measurements, creating personalised approaches to health and wellbeing. Opportunities exist to make individuals the primary unit of analysis. Future research should explore holistic personalised approaches to health monitoring in buildings that analyse the individual as they move between environments.

## 1. Background

Global urbanisation is resulting in a paradigm whereby people spend the vast majority of their time within indoor environments [1,2,3]. Yet, prolonged exposure to these environments can have a profound impact on health and wellbeing. Respiratory problems, headaches and skin conditions are some of the many symptoms that can be caused by poor indoor environment quality (IEQ)a term commonly used to describe the measurement of environmental parameters including air quality and visual, thermal and acoustic comfort [4].

### 1.1. IEQ, Health and Wellbeing

There is a growing body of research evidence [3,4,5,6,7,8,9,10,11] recognising the intricate interactions between IEQ, health and wellbeing which may be attributed to the complexity of parameters being measured. IEQ is not one single measurement factor, nor is there one single building design element that affects health and wellbeing, the latter actively promoted by many green building standards, (WELL building standard [12], Leadership in Energy and Environmental Design (LEED) [13] and Building Research Establishment Environmental Assessment Method (BREEAM) [11]). It is recognised that IEQ, health and wellbeing studies exacerbate the issues by inadequately communicating the causes and outcomes of wellbeing [14]. This makes it difficult to understand which environmental parameters lead to health issues and how to measure them.

Poor IEQ can lead to visible health symptoms. It can also affect cognitive abilities and productivity [15,16,17]. The impact these non-visible symptoms have within a workplace are drivers of IEQ research within commercial buildings. Yet, the indoor environment of all buildings can affect health and wellbeing and there is research that now focuses on IEQ within residential buildings. Inevitably, there are ethical, political and methodological difficulties, which make residential building studies more challenging than commercial [18].

### 1.2. Residential vs. Commercial

There are several reasons why implementing residential IEQ studies in real-world research is challenging. Residential buildings often lack suitable infrastructure, such as IT systems, building-wide environmental controls and building management systems (BMS) that make environmental monitoring routine. Furthermore, residential research lacks many drivers found in commercial building studies, e.g., employer responsibilities or corporate wellness programmes (which provide direct returns on investment if they are used to maintain workplace productivity [19]), cost and complexity of equipment [20]. To monitor multiple buildings simultaneously, more monitoring equipment is needed, which may be indicative as to why lower sample sizes are commonly reported as limiting factors in residential building studies [21,22,23].

Studies in open-plan offices benefit from being able to observe large sample sizes, monitored simultaneously with a small number of sensors. Commercial building studies also leverage state-of-the-art sensing equipment, which has implicit validity [24], acting as ‘reference standards’. Typically, those are built into many heating ventilation and air conditioning (HVAC) systems or retrofitted to existing systems. However, in buildings without mechanical ventilation, there is a requirement to place in-situ sensors in each room. This creates more measurement points and increases the capital investment required for environmental monitoring.

In the UK a substantial majority of residential properties are heated through hot-water central heating systems that use boilers and radiators [25] reducing the need for mechanical ventilation. Consequently, indoor environment measurement must be conducted using in-situ measurement devices. Furthermore, it is unlikely that participants of residential studies will all live in the same enclosed space; implying individual monitoring would be required.

### 1.3. Rationale

In research and laboratory studies, it is feasible to use small sample sizes and smaller spaces to reduce the amount of IEQ monitoring equipment required, reducing costs. Beyond research, there is a need for monitoring solutions to be scalable, enabling monitoring of buildings with many occupants. Unfortunately, promoting the use of state-of-the-art sensors on design projects is difficult due to the cost and equipment complexity, limiting scalability [7]. Consequently, many are exploring low-cost sensor solutions. Those range from consumer-grade, designed for immediate integration into smart-homes, to ‘do it yourself’ (DIY) devices that make use of low-cost sensing components as well as microcontrollers and single-board computers such as a Raspberry Pi. Nevertheless, the lack of scientific validation in these devices means many studies still require the use of expensive, reference-standard equipment as a baseline/reference measurement [7,21,26,27,28].

IEQ measurement is also limited due to subjective data gathering. It is common for thermal, visual and acoustic comfort monitoring to involve measurements of an occupant’s perception rather than just an empirical assessment of the environment itself. This leads to many discrepancies across the literature as individuals respond differently to changes in the environment [29]. Additionally, occupants are not always aware of what constitutes a poor environment, reporting issues that are directly observable only. This leads some to question the validity of subjective responses [30] but it is important not to discount that data. For a system to be effective in improving wellbeing and comfort, it needs to monitor the environment and measure how occupants cope and respond to environmental changes [31]. Objective measurements of individuals may be able to reinforce and possibly even substitute the subjective nature of occupant comfort measurements [32].

### 1.4. Objectives

The main objectives of this review are to:

1. Understand IEQ and how it is currently measured;
2. Examine sensor technologies used to capture IEQ;
3. Explore approaches to measure IEQ, identifying trends for monitoring occupant health and wellbeing.

## 2. Methods

This inquiry presents a scoping review (ScR) of studies that compare and evaluate sensor technologies used for measuring IEQ in buildings using the PRISMA-ScR methodology [33] checklist provided in the Supplementary Material (Table S1).

### 2.1. Searching and Selection Strategy

The initial phase of the review involved identifying keywords and filters that would be used to build search terms. The key filters used in this review are shown in Table 1. Combinations of filters were used to build search terms; for example, ‘(#2 AND #7 AND #14)’, which equated to ‘((IEQ OR “Indoor (Environment OR Environmental) Quality”) AND Sensors AND Building’. All queries were joined with an AND clause and OR was only used as indicated in Table 1. Literature was selected initially based on the inclusion of keywords within the titles. The abstracts and findings were then subsequently scanned to identify suitability to the aims and purposes of this review. Building type was also factored in when selecting studies. Focus was on residential and commercial buildings, but educational building studies were also included, given that mix of open-plan and enclosed spaces provides parity to office-based studies. Laboratory studies that analysed the technology were also selected to gain an understanding of the benchmarking process of environmental monitoring devices.

### 2.2. Eligibility Criteria and Information Sources

Given the nature of emergent technology, the scope of this inquiry was limited to publications within the last ten years and to literature, which directly used hardware to monitor IEQ and not surveys and occupant feedback only. The primary source of information was peer-reviewed academic journals and conference proceedings and multiple databases were used to search for literature, including; ScienceDirect, Scopus, PubMed, IEEE Xplore and Google Scholar.

### 2.3. Charting Screening and Synthesising Data

Data extracted from selected studies were collated and presented. Table 2 provides an overview of building types, environmental factors and the types of technology used to measure them. Where possible, demographic details were also extracted. In total 27 papers were reviewed. Data on measurement devices were subsequently presented in Table 3 and Table 4, which identify specific environmental factors that were measured. Table 3 outlines state-of-the-art sensors, which are devices that are used throughout the literature as a reference standard to either validate low-cost sensors or as standalone measurement devices. Table 4 outlines low-cost devices, electronic components that can be incorporated into DIY monitoring devices. An exception was the Netatmo Weather Station, because it is cheaper than some of the more expensive DIY components such as the GSS COZIR or the Sensorist Wireless Pro T/RH and also because it requires calibration against a reference standard [34]. In total, 33 state-of-the-art and 28 low-cost devices were identified across the 27 papers. The sensors that were used indicated that there is a prevalence of IAQ and thermal comfort across the studies but with many inconsistencies relating to measurement. Throughout the review, the data will be used to communicate the technologies, methodologies and findings from the selected studies and their relationship to state-of-the-art and low-cost environmental sensing. The reader should use Table 2, Table 3 and Table 4 for reference when reading the data syntheses, as these tables categorise studies according to key data items, technologies and demographics.

## 3. Understanding IEQ and How It Is Measured

IEQ measurement comes in two forms: (i) measurement of physical environmental changes that can be quantified using objective monitoring equipment and; (ii) subjective data on how occupants perceive indoor environments, via surveys or self-reporting which is referred to as ‘comfort factors’ [5] *(the reader is directed to that study for a very clear outline of comfort determinant types that are present within offices and residential buildings).* Hanc et al. [14] highlight the importance of clarity surrounding wellbeing and note that environmental studies often fail to make clear distinctions between outcomes and determinants. They note ambiguity between comfort, satisfaction, and wellbeing, found in many environmental studies, exacerbates this issue. This is problematic, as it prevents researchers and practitioners from being able to accurately compare studies to ask meaningful questions of IEQ. It also makes it difficult to determine what methods and research design models should be applied when attempting to measure IEQ, health or wellbeing in future research.

Many environmental factors can be measured quantitatively and there are many measurement devices available for this. However, given the complexity of IEQ, it typically cannot be defined by a single outcome, though some have tried to encapsulate it in the form of an IEQ index [39,44,48,49]. Tiele et al. [39] use IEQ-Index as a term to measure a range of environmental factors, including temperature, humidity, carbon dioxide, volatile organic compounds (VOCs), carbon monoxide, illuminance, sound levels and particulate matter less than 10 micrometres in diameter. Others [48] use the term *I_IEQ_* to encapsulate the sample mean from the sum of indoor air quality (IAQ) index (*I_IAQ_*), thermal comfort index (*I_th_*), visual comfort index (*I_v_*) and acoustic comfort index (*I_a_*), according to the following equation:

(1) $$I_{IEQ}=\bar{\chi }=\frac{\left(I_{IAQ}+I_{th}+I_{v}+I_{a}\right)}{\eta }$$

**Figure 1:**
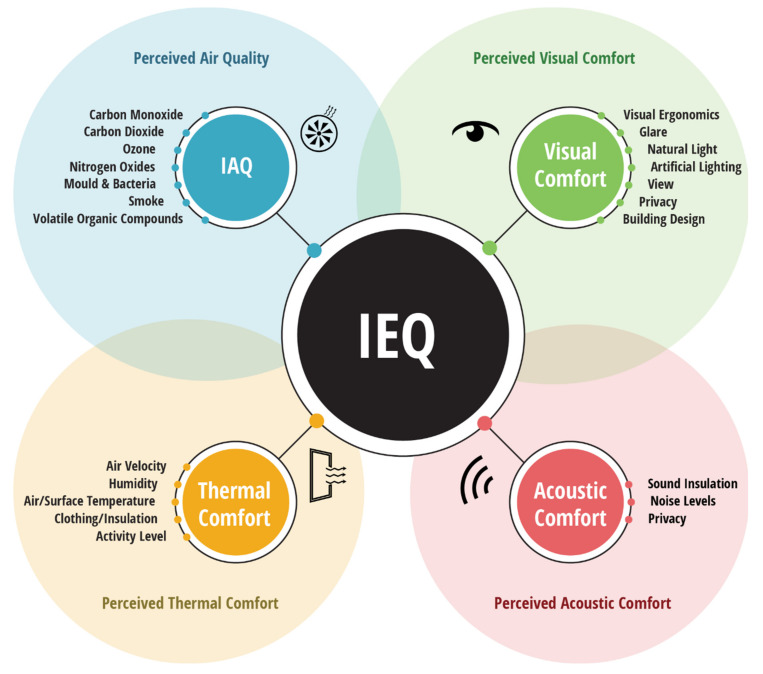
A holistic and general capture of terminologies and themes used across the literature to discuss IEQ.

It is suggested that this number could be used, as a ‘star rating’, but comparing indices used in separate studies [39,48] would be futile, given the two IEQ indices are measuring different environmental factors. Since there is no standardised approach to IEQ indexing, it is likely to further obfuscate the subject area and complicate inter-study comparison. The complexity of IEQ is seen throughout the literature as researchers attempt to provide their own in-depth overviews of what constitutes IEQ, where many authors accept and state that IEQ constitutes four key objective and subjective sub-factors: IAQ, visual comfort, acoustic comfort and thermal comfort [3,5,29,48,50]. Figure 1 highlights these factors and sub-factors of IEQ and demonstrates the relationships between Perceived Environmental Quality factors, which are highlighted in the underlying Venn diagram. The determinants of each sub-factor are also included, but these are what are commonly quoted rather than an exhaustive list.

### 3.1. Indoor Air Quality

**Figure 2:**
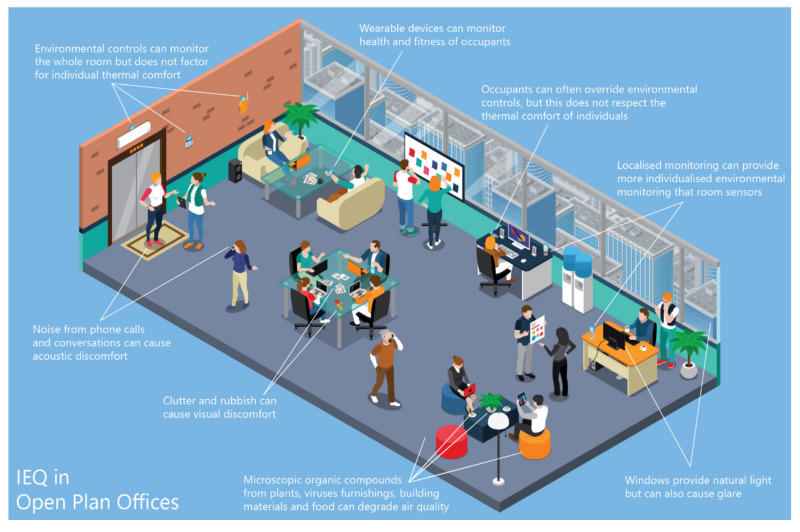
In Open Plan Offices.

Except for subjectively measured perceived air quality (PAQ), most air quality measurements can be done objectively. Table 4 shows some of the measurable indoor pollutants that contribute to poor air quality. Common factors of air quality seen across the literature are CO_2_ and VOCs which are fine breathable particles that are distributed into the air from building materials, food, viruses and furniture [51] (Figure 2). Many air quality studies [20,23,26,34,38,39] measured IAQ from particulate matter and/or VOCs but the most commonly measured factor of air quality across the literature is CO_2_ (Table 3 and Table 4). Probably due to the impact it has on workplace productivity as opposed to its impact on health.

#### 3.1.1. Carbon Dioxide (CO_2_)

Health conditions attributed to CO_2_ are not commonly present when exposed to less than 10,000ppm and CO_2_ concentrations under 5000 ppm are considered safe for eight-hour exposure [52]. Whilst not immediately threatening to health, exposure to CO_2_ levels above 1000 ppm can have an impact on cognitive functioning, productivity and comfort [34].

Allen et al. [34] monitored CO_2_ in offices for two-weeks using an off-the-shelf Netatmo Weather Station, calibrated to a reference-standard TSI Q-Trak sensor. Data were supported by surveys and self-reported sick building syndrome (SBS) symptoms, defined by The World Health Organisation as reported health-related symptoms that are caused by poor IEQ [53]. After the initial two-week period, participants were relocated to a building certified as Platinum by the LEED green building standard [13]. The study found participants reported 43% more SBS symptoms when the CO_2_ levels rose above 1000ppm. However, authors note that participants were aware of the test conditions including relocation details to a high standard ‘green building’. Participants reported more SBS symptoms when they were in a ‘non-green building’; even when environmental conditions in the building were optimal. Given how subjective occupant perceptions are, passive sensors can be an important way to reinforce findings through objective measurements.

Shan et al. [45] found links between CO_2_ and SBS. Their study monitored air quality and thermal comfort of two rooms using a range of state-of-the-art sensors (Table 3). Thirty-nine participants completed self-reported SBS symptom and thermal comfort questionnaires. Additionally, participants completed a series of tests that would evaluate their cognitive abilities, whilst air quality measurements were conducted. Authors found inverse correlations between cognitive performance and CO_2_ concentration levels with CO_2_ to be the main cause of SBS symptoms. Those authors suggest that since CO_2_ concentrations correlate with SBS symptoms, it is possible that higher CO_2_ concentrations attributed to decreased performance because participants were also experiencing discomfort. Their study also identified correlations between CO_2_ and other airborne contaminants, making it difficult to establish definitive causal links between their outcomes and CO_2_ concentration levels. Some other studies suggest CO_2_ is an inadequate measure of IAQ [20,38,54] and airborne contaminants such as particulate matter and VOCs are a more valuable indicator of IAQ. However, CO_2_ concentrations are known to increase when Air Exchange Rates are reduced [20,35]. This may indicate why increased CO_2_ is found to correlate with concentrations of airborne contaminants.

#### 3.1.2. Airborne Contaminants

Particulates and VOCs are known to accumulate within indoor environments and are regarded as a great environmental risk to health [8]. Building standards such as LEED and BREEAM, provide guidance and accreditation for the management of IAQ. However, only a small amount of accreditation points are awarded for it so there are insufficient incentives to encourage the additional work [55]. Alternatively, energy performance is often more valuable, but studies [21,22,23] show that reduced airflow and increased air-tightness required to increase energy performance, results in the concentration of contaminants and a reduction of IAQ.

Coombs et al. [21] investigated non-green, multi-residential apartments home to asthmatic children (7–12yrs). The inquiry was conducted as buildings were renovated to comply with green building standards. Airflow and IAQ were monitored in eight homes before and after the renovations using the reference standard SKC AirCheck 2000. Air filters were attached to the latter in order to collect airborne contaminants. As a control, IAQ was simultaneously monitored in a low-income, non-green, multi-residential complex. Authors discovered significant differences in properties before and after renovations and found reduced airflow and increased airtightness, typically required to increase energy performance, resulted in an increased concentration of contaminants and a reduction of IAQ. Similarly, Broderick et al. [23] monitored fifteen, three-bedroom, semi-detached social housing properties. Their study measured airborne contaminants (not airflow) using a range of state-of-the-art sensors (Table 3). IAQ was monitored in the living room and master bedroom of each property before and after an energy performance renovation. Their study revealed an 18%–25% increase in CO_2_ and VOC concentrations levels and a 40% increase in the concentration of particulate matter up to 2.5 μm in diameter (PM2.5) after buildings were renovated. Findings also revealed negative correlations between energy performance and air quality. Furthermore, whilst CO_2_ may not provide an adequate determination of IAQ, there are links between PM2.5, VOCs and CO_2_ and those links may explain the prevalence of CO_2_ in IAQ studies across the literature [21].

### 3.2. Thermal Comfort

IAQ is not a standalone factor of environmental quality, being influenced by many other objective and subjective IEQ factors. Occupants have been found to report poor PAQ when they are thermally uncomfortable [56], explaining why many studies focus on IAQ and thermal comfort. ISO 7730 [57] defines thermal comfort as being associated to a person’s thermal balance and is affected by clothing, physical activity, temperature, humidity, movement of air and the average temperature of surfaces in a room (mean radiant temperature, MRT), Table 4. Prevalence of thermal comfort across the literature may be due to its intrinsic influence over PAQ, but it may also attribute to the maturity of building standards that focus on thermal comfort. Those standards specify thermal comfort factors and how to measure it. This standardisation of measurement means that thermal comfort studies can be directly compared.

#### 3.2.1. Predictive Mean Vote (PMV)

The ASHRAE Standard 55 widely measures thermal comfort [4,32,36] and should be done using occupant satisfaction surveys, point-in-time surveys and electronic sensors measuring the thermal environment [58]. ISO 7730 and ASHRAE 55 standardise a predictive mean vote (PMV) steady-state model [59] used for measuring thermal comfort. It does this by predicting occupants mean thermal perception and predicts the percentage of those who will be dissatisfied by the thermal conditions [60]. However, it was found that this approach did not accurately predict thermal comfort [61], which may be attributed to the fact it was not field-tested before being incorporated into the standard [62]. The model has also been criticised for its ineffectiveness in naturally ventilated buildings [32,63], it does not account for climatic differences and occupants are given little to no control over their own thermal comfort.

#### 3.2.2. Adaptive Comfort Model

To challenge concerns around the PMV model, an adaptive model was developed [63], which acknowledged that occupants in naturally ventilated buildings have a much broader tolerance threshold for thermal conditions than those in mechanically ventilated buildings. That model is recognised in British Standard EN 16798-1 [64] as well as the deprecated British Standard EN 15251 [65]. De Giuli et al. [4] utilised the latter standard, prior to its deprecation, to assess whether children would be able to perceive environmental changes in non-mechanically ventilated schools. This was measured according to the ISO 7730 standard [57] using a Brüel and Kjaer climatic analyser. De Giuli et al. [4] found that children were able to perceive poor air quality and noise, perceiving poor thermal comfort in the summer. They also noted that since the environmental conditions of the classroom were set according to the preferences of the teacher, children were found to be unaware of many conditions or behaved as passive users of the environment. This was found to be the case in all schools other than mechanically ventilated schools where students showed they were more aware of the environmental conditions and they had more control over it.

#### 3.2.3. Occupant Control

It is believed that occupants should be given control over mechanical ventilation systems [32], as environmental control plays a role in personal comfort [66]. Li et al. [32] found that environmental studies were often limited by ventilation systems that lack the capabilities to facilitate such control. However, providing user access could lead to dissatisfaction due to individualised perceptions of comfort (Figure 2) but too much control can also distract workers from their duties [67]. To overcome these issues, Li et al. [32] designed a system which allowed participants to use a smartphone to vote on how comfortable they are and provide details of their clothing and level of activity. This allowed occupants to have control over their environment, without control becoming a distraction. The smartphone application also collected data from a COZIR CO_2_ sensor, a sensorist wireless pro temperature and humidity sensor. Data were collected and combined with participant votes and were used to alter the HVAC thermostat set point. To test, authors conducted studies in single-occupancy rooms and in an open-plan space. By substituting static-set point thermostats with their system, they found that reports of thermal discomfort dropped by >50%. Inclusion of physiological data also serves to remove much of the subjectivity from traditional thermal comfort measurements. However, their study failed to take air velocity or MRT into account and instead measured CO_2_ as a determinant of IAQ, but since their system was developed to take data from multiple sources, it is likely that it could be adapted to include measurements of air velocity and MRT.

### 3.3. Visual Comfort

Visual comfort is highly influential on other subjectively measured environmental factors. Table 4 shows some visual comfort factors and highlights its influence over PAQ and acoustic comfort. Although there are many subjective visual comfort factors, light intensity or illuminance, measured in LUX (lx) is the major objective measurement (Table 2). The threshold for illuminance is dependent on task but for most office tasks, thresholds range from 300l× to 500l× [68]. Yet, ambient illuminance will likely not reflect the light levels at individual workstations. Light reflectance and glare can cause areas of visual discomfort (Figure 2), but the ambient illuminance can be within specified limits.

Many green building standards recognise visual comfort extends beyond base level illuminance and are establishing new parameters of visual comfort. The WELL Building Standard includes a range of factors including glare control, fenestration of daylight, ergonomics of the space design and lighting colour [12]. All but one study measured visual comfort using LUX only (Table 2). The exception [36], measured visual comfort using a combination of LUX and a measure of lighting colour, using the Wovyn Color Lux1000. Their approach measured the impact of blue light on sleep but also the effectiveness of window tinting on a room’s ambient colour temperature by placing light sensors at desk level and in elevated positions. This allowed authors to identify the environmental variability of daylight and artificial lighting in buildings. They acknowledged that studies in controlled environments could mitigate this variability, but felt their study provided more natural conditions. By including RGB sensors they identified many key aspects of visual comfort identified by the WELL Building Standard [12].

### 3.4. Acoustic Comfort

Noise is a major contributor to discomfort in many naturally ventilated buildings [67]. Noise can come from a number of sources, but any sound that causes distractions to everyday actions, such as relaxation or work, can be considered noise in the context of occupant comfort [69]. A few studies [7,16,20,41] measured noise using a microphone, which provides a measurement of sound pressure level (SPL) in decibels (dB) (Table 2). According to the ASHRAE’s guidelines [70], the sound levels in open-plan offices should not exceed 45dB [71]. This is closely mirrored by the WELL standard for sound masking in those spaces [12], which states that levels should not exceed 48 dB. Whilst those standards provide strict limits on noise levels, they do not translate to acoustic comfort. In offices, noises often come from mechanical or electrical equipment, conversations or phone calls from surrounding occupants [36,72] (Figure 2).

Notwithstanding the fact that office noises can be a great source of discomfort for building occupants, they are often well within the specified limits. As noted by Tiele et al. [39], this makes SPL measurement ineffective at measuring acoustic comfort, as levels of perceived noise may not match those captured by electronic equipment. Moreover, they indicated that there are more quantitative measurements that should be considered in research, such as sound variations and peaks. Even with objective methods to support measurement, acoustic comfort is predominantly subjective. With one-in-six people in the UK suffering from hearing impairment [73], this subjectivity must be assessed on a case-by-case basis. Unlike thermal and visual comfort, acoustic standards specify the thresholds of the objective measures and do not provide a standardised approach to measuring occupant perceptions of their acoustic environment.

## 4. Understanding State-of-the-Art Environmental Monitoring

BMSs control and manage building assets such as HVAC systems [74]. These systems are typically used by facility managers for scheduling asset maintenance but extend to the collection, storage and transmission of asset data using built-in state-of-the-art sensors. HVAC systems are often retrofitted or preinstalled with sensors that monitor air quality, temperature, humidity and flow. Using a BMS to monitor assets is considered a well-established approach that provides useful data [24]. Occasionally, this may not provide a useful monitoring solution when supplying air to multiple spaces. Generally, air distributed in each space can be monitored with a single measurement point, but this does not always provide an accurate portrayal of the environmental conditions experienced by occupants [20]. Handheld monitoring devices may provide a better-individualised approach.

### 4.1. Data Loggers

Five studies referenced BMSs and HVAC systems, most of which used state-of-the-art monitoring devices in commercial buildings. However, there are many types of buildings that do not have the supporting assets to warrant using a BMS, such as single-family residential buildings. In those, environmental data can be collected using data loggers, which are portable monitoring devices with built-in storage. Several studies investigated IEQ within multi-occupant spaces using state-of-the-art data loggers [4,24,27,44] (Table 3). Typically, most are designed to be handheld for point-in-time measurements or periodically mounted within buildings for continuous monitoring.

The most common data logger manufacturers were TSI and GrayWolf with prices ranging between a few hundred (Onset Hobo U12-012) and to several thousand pounds sterling (TSI DustTrak 8532). Whilst the accuracy and precision of these can make them extremely valuable tools, there are many drawbacks making them un-pragmatic. For example, with the Extech SD800 or the Wholër CDL 210, data are stored within internal memory and later downloaded. Therefore, real-world applications are limited to point-in-time measurement or short-term studies such as post occupancy evaluations (POEs).

Primarily, a POE is the process in which buildings are evaluated, after the point of occupancy, to assess whether the building performs according to the occupants’ needs [75]. POE also focuses on post-construction building performance to assess whether it meets design specifications [76]. When buildings are designed according to standards that specify IEQ thresholds there is a need to measure environmental factors to ensure it meets those standards after occupation, typically running for two to eight weeks [77,78]. Whilst data loggers of this type may be ideal for conducting such evaluations, they lack the ability to provide real-time feedback making them impractical for continuous environmental monitoring.

### 4.2. Scalability Limits around State-of-the-Art Solutions

Open-plan office studies often mitigate the state-of-the-art cost by measuring multiple participants in a single location, as fewer sensors are needed to measure the space and larger sample sizes can be observed. However, since residential studies measure participants across multiple properties, small sample sizes [32,36,41] and short measurement periods [4,22,23,40] are often built into the research design to address budgetary restrictions. McGill et al. [22] measured air quality in buildings that were built according to the German Passivhaus standard, an approach using passive design systems to maintain a balance between environmental quality and energy use [79]. They measured air quality using state-of-the-art data loggers from Extech and Wholër. However, the measurement period was short and the sample size was both limited and split across multiple buildings. This resulted in findings that can only be used to provide insights.

Contrastingly, a commercial office study [36] measured a similar sample size but because participants were within the same environment, sensors could be used to simultaneously measure multiple occupants. Large portions of the building could also be monitored more easily; however, authors note this made it difficult to provide individuals with paralleled IEQ environments. However, the cost of the equipment used in this study means that it is highly unlikely that this methodology could be applied outside of research.

An emergent market of low-cost accessible devices is available, which opens use cases that can drive the future of research, whilst addressing capital investment requirements. It is, therefore, important to understand and research where low-cost technologies can add value as standalone measurement devices for both researchers and practitioners.

## 5. Low-Cost Alternative Technologies

Low-cost microcontrollers and microcomputers (e.g., Arduino and Raspberry Pi, respectively) are becoming valuable IEQ measurement tools. Many devices use open-source hardware, which advances technological development through a community [80]. This approach means users can become developers, instead of consumers. Furthermore, open-source hardware actively permits the creation of clones, which are cheaper alternatives to the official products or devices that are modified for a specific use [81]. Many of those sensors are now also being incorporated into “breakout” boards, which are low-cost, universal devices designed to interface directly with a serial bus on microcontrollers and microcomputers [82]. This means that technology used to monitor IEQ is becoming accessible, easier to develop and can be significantly cheaper than state-of-the-art counterparts [43]. Prevalence of DIY devices (Table 2), is testament to a paradigm shift that is breaking down IEQ monitoring entry barriers.

### 5.1. Limitations of Low-Cost Sensors

Low-cost sensors also use cheaper components than state-of-the-art equivalents. For example, CO_2_ sensors typically use infrared to detect gas concentrations. Sensors such as the MH-Z1x range and CozIR are cheaper alternatives to state-of-the-art CO_2_ sensors, such as the Wholër CO2 Data Logger. Whilst all infrared CO2 sensors measure CO2 in the same way, cheaper components are used in low-cost sensors. Other low-cost sensors, such as the CCS811 and the iAQ-Core C, use different technology all together by detecting gasses that come into contact with a semiconductor that has a metal oxide surface [83]. Those sensors typically provide a CO_2_ measurement; though not actually a measure of carbon dioxide (Table 3). In fact, data from the sensor measures the total concentration of VOCs in the air (TVOCs) [84] which is returned at a different scale factor known as equivalent carbon dioxide (eCO_2_). This approach lacks transparency as none of the datasheets [85,86,87] (Table 4) articulate how eCO_2_ is calculated, nor do they state whether a standardised calculation method is used. Therefore, it would be difficult to distinguish whether any differences in measurements were caused by the conversions or the sensors. Furthermore, a lack of clarity about what eCO_2_ is and how it is calculated has led some to consider it an actual measure of CO_2_ [88,89]. Issues such as these are likely contributing factors to mistrust with low-cost devices.

### Accuracy vs. Precision

Low-cost sensors have been found to be less accurate than state-of-the-art sensors [20] but they have been found to have good precision [83]. This means they may not provide an accurate measurement of environmental factors but will be responsive to changes. For example, if a room contains a CO_2_ concentration of 650 ppm and the CO_2_ rises by 10 ppm/min, an inaccurate but precise sensor may read an initial value of 900 ppm, but still, measure 10 ppm concentration increases. Ultimately, the suitability of a device depends on the application, as a low-cost device would be unsuitable where precision and accuracy is needed for a building to ensure it meets a government regulation. Conversely, high-accuracy, low-precision devices would be suitable for studying CO_2_ elevations on the concentration levels of occupants.

### 5.2. Scalability

Mihai and Iordache [44] highlight how cost can affect IEQ research. In their study, a single (£1500) CALCTM 7525 was used to measure air quality in university classrooms. Measurement of illuminance was conducted using an array of nine Lurton LM-8102 light meters (£100/each) per room, placed at each student’s desk. Whilst the Lurton sensors are reference-standard equipment, the price difference of the sensors is indicative of the level of granularity in the measurement of the two IEQ factors. The effects of the inconsistencies in measurement granularity can be seen in the visualisations provided in their article, which affected their findings. Each index of IEQ was mapped to the floor plans of the building. IAQ, thermal comfort and acoustic comfort were measured and visualised on a room-by-room basis, where one room may perform better or worse than another. However, visual comfort was measured and visualised at an individual level, meaning certain areas of a single space were found to perform better or worse than others. This is significant as visual comfort was measured at an individual level only. The combined IEQ index was visualised using the buildings floor plans and their visualisation clearly highlights how data were skewed by the visual comfort measurements. The study serves as a good indicator of how scalability can affect study design, whilst clearly highlighting the value of the localised measurement.

Mihai and Iordache [44] measured environmental factors at an individual level but devices could be termed state-of-the-art and considered as relatively expensive when compared to other light sensors (Table 4). Notwithstanding measurement accuracy, low-cost devices may be more suitable for measuring how individuals are affected by environmental changes. By using such devices, it is possible to incorporate more sensors at a very low cost, which will allow measurement resolution to be increased and focused on the individual. To make this increased resolution scalable, there is a need to use a holistic system like a BMS to capture, record and analyse the data from multiple, different sensor sources but such systems are not always available or applicable.

### 5.3. Holistic Cloud-Based Systems

Cloud-based platforms are rapidly increasing in popularity and are often inexpensive, open-source or are delivered as a scalable service but require internet-enabled measurement devices. Reference-standard, portable data-loggers are typically offline devices that store data. Wireless data loggers exist, but often interface with proprietary web platforms only and are more expensive [24]. Most of the low-cost sensors in Table 4 are not standalone wireless devices. Instead they are sensor components that need to be connected together using microcontrollers or microcomputers such as Arduino or Raspberry Pi. Once connected together, these devices can read and write data from sensors either to local SD card storage [39,43] or transmitted wirelessly to cloud platforms [6,7,32,34,38,40,46,47]. However, there are several approaches seen across the literature to bridge the gap between the device and the cloud.

Across the literature in Table 4, there are three approaches for wirelessly transmitting data from DIY sensors, the first approach simply involves using WiFi enabled sensors in the first instance [32,34]. However, the second and third approaches involve developing hardware devices with WiFi capabilities and there are two approaches seen across the reviewed literature for doing this. One method involves using wireless sensor networks (WSNs), which are a network of wireless devices (nodes) that connect to each other to form a network that enables data transmission over large distances with low power consumption [90]. This approach was found to be advantageous, as it facilitated the simultaneous collection of data from different devices through the various nodes [6]. However, additional to the sensor nodes, there is often a requirement for the network to contain access points and gateways, which the nodes must first communicate with [38]. Contrastingly, modern microcontrollers now come included with on-board WiFi chips [7,38,40,47], which allow the devices to directly communicate with wide area networks. WiFi shields can also be used to add-on wireless functionality to boards that otherwise would not have it [46]. This approach also has benefits as it removes the need for gateways and hubs, potentially reducing project costs.

Since many of these sensors are integrated into custom-made devices, they do not depend on proprietary systems to visualise or analyse data. This means developers have the freedom to connect to a wide array of cloud-based applications or create custom architectures which is reflected in the literature as no two studies implementing web-based platforms [6,20,32,35,36,38,40,41,46] used the same web architecture or visualisation platforms. Need for wireless monitoring has created a competitive market for cloud-based applications and interactive dashboards to display sensors data. Consequently, there is no one standardised approach seen across the literature for storing, recording and analysing sensor data.

Cloud-based applications allow the creation of complex rules and associations [91], meaning that sensor data can be concurrently associated with a building, a room and an occupant. That process can be streamlined and improved by incorporating data from 3D models containing building information modelling (BIM) data, as shown in a recent study [35]. Those models contain a wealth of information about buildings including spatial structures and asset information. These data can be integrated into a holistic system that collects data from multiple sources including environmental sensors and subjective occupant feedback. This makes it feasible to monitor individual environments with a wide range of sensors and understand how building design and environmental changes impact occupant health and wellbeing.

## 6. Individualised IEQ Approaches for Health and Wellbeing

Compared to health outcomes measurement of wellbeing can often be challenging given the subjective nature of what is being measured and lack of a standardised method for which to collect data. Moreover, the wording of questions, ambiguous responses and inconsistent administration techniques mean that there are many limitations with these measurements [92]. Furthermore, it is common for studies on wellbeing and the indoor environment to lack clarity in the methods used to collect subjective wellbeing data. For example, the clarity of questions is not explicitly detailed and/or there are unclear links to wellbeing outcomes [4,9,93,94]. Yet, whilst ambiguity around research design does not invalidate findings, repeating studies or identifying patterns across the literature is challenging. Therefore, it is difficult to understand whether IEQ measurements have the efficacy of determining wellbeing. Given the tenuous nature of links between IEQ and wellbeing, there may be value in exploring links between IEQ and health, as good health is found to positively impact wellbeing [95].

### 6.1. Holistic IEQ Approaches

Researchers have discussed the prevalence of using sensors to monitor the relationship between occupants and their environments [6,7,8,9,10,40] but few make the individual the primary unit of analysis. Measuring individual response to changes is a key factor of occupant comfort and described as an important requirement for environmental monitoring systems [31]. It is proposed that non-invasive, wearable, health-and-fitness technologies are an accessible way to monitor a range of psychological and physical health conditions such as depression and hypertension, respectively [96]. These technologies enable researchers to access a vast repository of individualised health biomarkers, which could be used to augment passive IEQ measurement by using smartwatches, smartphones, smart-clothes and even smart-tattoos [97]. Three studies [16,32,36] used personal fitness trackers to monitor a variety of health data in relation to IEQ. These studies all involved the collection of data from multiple sensors and they each highlighted potential links between occupant physiology and IEQ. Moreover, the methods used in these studies highlighted the need and value of holistic IEQ approaches.

In recent years, wearable health and fitness market has become saturated with new devices, so it is not always clear which devices are most appropriate where many are released and discontinued each year. A recent review [98] highlighted that personal fitness trackers (PFTs) such as Fitbit and Garmin feature heavily across the literature, providing a checklist, which outlines eight categories to appraise PFTs, a useful starting point for those considering the use of PFTs in research projects. The wearable market seems to have split consumers into those who want expensive smartwatches that integrate with smartphones and those who want lower-cost PFTs, such as devices by Xaiomi and Huawei. The latter is driving down PFT cost, which means they could be built into scalable monitoring tools.

Many of the applications of PFTs across the literature involve either evaluating the gamification of health or looking at the effects PFTs have on daily routines. Measuring daily steps can have a positive effect on health, as it is indicative of a more active daily routine [99]. This can also serve as an objective measurement of activity levels that can be used to support IEQ studies. Though it is important that users are actively involved in the early stages of health technology research and design to ensure the technology is developed and appraised with a user-centric approach [100]. Moreover, it is important that during this process users are made aware of how their data will be collected, stored and analysed to ensure it is compliant with data protection standards (for example, the General Data Protection Regulation (GDPR)) and is done so in an ethical manner.

### 6.2. Linking Health to Wellbeing: Augmenting IEQ Approaches

Continuous in-situ measurement via wearables has the potential to provide individualised health measurements, augmenting IEQ approaches by providing quantitative data to support qualitative data, by reducing errors found in subjective measurements of wellbeing [101]. MacNaughton et al. [16] combined wearable health data with data obtained from IEQ sensors and results from health surveys that assessed cognitive function. Whilst their study did not eliminate the need for subjective responses, the inclusion of the additional health data meant that additional insights were formed. The study found patterns between the quality of sleep and cognitive ability and associate the former with environmental conditions such as lighting. Moreover, by objectively monitoring the individual, authors were able to make definitive links between environmental factors and physiological responses.

Diminishing costs and increased accessibility of environmental sensing technology mean that scalable solutions can be developed that takes a personalised approach to wellbeing measurement. Localised environmental monitoring augmented with wearable data can be fed into holistic monitoring systems, providing meaningful results to both building owners and occupants. Augmenting wearables with low-cost IEQ approaches will allow research to extend beyond a single environment, as sensors can be placed in multiple environments, such as the home and workplace and wearables can be worn as occupants move between environments, but to compare different environments the data collected from each environment must be comparable. Unfortunately, studies show that approaches to environmental monitoring differ greatly between residential and commercial buildings, as does the technology used. There is a clear need for research that addresses these knowledge gaps by considering longitudinally IEQ measurements and individualised approaches to monitoring alongside within the home and workplace. This will provide a better holistic picture of how their physiology is affected by those environments. It will also allow researchers to draw conclusions about the impact buildings have on occupant wellbeing.

## 7. Discussions

This scoping review presents approaches to IEQ measurement in buildings from a range of research domains, whilst exploring technologies and approaches to health and wellbeing trends.

### 7.1. Understanding IEQ

It is evident that IEQ is a complex and multi-faceted area of study and efforts to define it often clutter the definition rather than add clarity. This is exacerbated further as researchers attempt to encapsulate IEQ measurements into a single IEQ-index [39,44,48,49], which could have a profound impact on future research. Researchers may be inclined to compare like-for-like, but this may lead to inaccuracies unless this encapsulation follows a defined standard. Currently, IEQ indices should be treated with caution until the literature provides a common understanding of what constitutes an IEQ index and which factors it encapsulates. It is also important, that researchers do not compare IEQ indices directly unless they are sure they are comparable measures.

There is a general acceptance that IEQ consists of four sub-factors: IAQ, visual comfort, acoustic comfort and thermal comfort. This may suggest that Equation (1) [48] is an appropriate index. However, there is further confusion about what constitutes each of those sub-factors. Given the current state of literature, it is not possible to gain a definitive understanding of what factors constitute IEQ, but this review has highlighted many of the factors, which have been measured. This list is not exhaustive and the way in which IEQ is measured is inconsistent and conflicting across studies. Table 3 and Table 4 highlights only a snapshot of the environmental factors that make up the sub-factors of IEQ.

There is little commonality across the literature over the quantity or combinations of environmental factors that must be measured in order to satisfy a measure of air quality or thermal comfort. For example, Tiele et al. [39] measure IAQ with a single measure of CO_2_. Whereas, Li et al. [32] measure IAQ with multiple measures of VOCs, Carbon Monoxide, CO_2_ and particulate matter. Whilst this may suit the needs of the individual inquiries, it adds complexity to the subject area. It is pertinent that future research aligns to a common understanding of what constitutes IEQ. However, more needs to be done to standardise and legislate a homogeneous IEQ measurement. Notwithstanding the confusion about what should be measured, there are a plethora of environmental factors that can be measured, using environmental sensors.

### 7.2. Understanding IEQ Measurement Technology

This review highlights the range of devices used and outlines how state-of-the-art monitoring devices compare with low-cost sensors. The primary differences between these devices are cost, accuracy and connectivity. Since the cost of state-of-the-art sensors is prohibiting and difficult to promote on projects [7], it is important to understand the needs of the project before procuring hardware. It is also important to understand how the research is to be applied. If the purpose of the research is to benchmark low-cost sensors [26], then it is feasible to use reference-standard equipment to act as a baseline for measurement. If the study is proposing solutions that could be adopted by practitioners [36], it is not feasible or pragmatic to propose such expensive equipment.

This review found that the accuracy and precision of devices are regularly questioned, particularly with low-cost approaches. Low-cost devices are found to have lower accuracy, but they have good precision [83]. However, whilst reference-standard devices may have higher accuracy in a laboratory test; in practice, equipment costs lead to fewer devices being used. This means it is not possible to gain an accurate indication of what individuals experience [20]. Using low-cost devices, it is possible to counteract the reduction in accuracy by increasing the number of measurements. It is feasible to measure individual environments so that data can be analysed more accurately alongside an individual occupant, but this results in a high degree of data points that need to be stored, visualised and analysed.

This review discovered that there is a rapid market growth surrounding low-cost hardware and cloud-based applications that these devices can interface with. Such applications are hardware agnostic meaning that any device that can send data could send it to a holistic monitoring system. This may bring the power of high-cost BMSs to small businesses and even individuals. Moreover, since data obtained by these systems are not just limited to building assets, it is possible that occupant surveys and even data from wearable sensors could be included in holistic systems. This makes it possible to understand how occupants respond to environmental changes within their immediate environment.

### 7.3. Augmenting Current IEQ Approaches

We found that wearable devices have demonstrable value to research in this area. Granular measurements can be taken at an individual level that can be used to support traditional environmental monitoring. Localised sensors can measure an individual’s immediate environment, but wearables could also be used to collect an individual’s psychological and physiological data, used to better inform or support findings. Wearable devices also have the potential to monitor for health biomarkers, which could make it possible to understand how individuals are affected by changes in the environment from quantitative data alone. By measuring individualised physiological data, Li et al. [32] were able to demonstrate a personalised approach to monitoring that provides a wealth of data to inform, reinforce or even replace traditional subjective measurements of comfort. However, research is needed to understand, which health biomarkers and wellbeing parameters correlate with each other. This may make it easier to quantitatively associate IEQ with wellbeing, but it will also mean that researchers must pay closer attention to the physiological conditions, as they will have a greater impact on individualised measurements than they would on group studies.

### 7.4. Limitations

ScRs have several limitations, due to the broader scope. One of the key limitations of ScRs is the lack of a mature framework for conducting the review [102]. It was for this reason that PRISMA-ScR was adopted to ensure there was rigour in the process. However, there were still limitations in this review from conducting the review as an ScR. The key limitations in this review were establishing the boundaries of the review and the time taken to conduct such a broad range of topics. It was easy to become overwhelmed by the literature, which spanned multiple research disciplines. Further, presenting such a broad range of subject areas, in a way that aligned with the objectives of this study, was a time-consuming process. This was further exacerbated by the need to iteratively search for new publications during that time. However, due to these limitations, it was not possible to capture all studies across such a broad scope of literature, whilst providing a concise and relevant analysis. Therefore, it is understood that there will be studies from each discipline that were not captured in this review due to the broad search strategy and the lack of a narrow scoped systematic analysis.

### 7.5. Future Research

There is a need for a paradigm shift that makes the individual the unit of analysis. The high percentage of time people spend indoors is not only spent in one single environment; yet, most studies only target a single environment, such as schools, offices, or homes. Researchers should take advantage of the scalability of low-cost devices, as it will enable them to incorporate more environments into their studies. Measurements could be taken at work, at home and even as they commute, to get a more holistic picture of each individual. However, few IEQ studies do this. Instead, human participants are typically found to provide contextual data to reinforce environmental data.

The confusion around IEQ and the factors of measurement are present, but there is a wealth of literature that can inform researchers to draw their own arguments and ideas on which to found their work. However, the lack of studies that monitor the environment from the point-of-view of an individual is a knowledge gap that must be addressed. In addition, given that there are recognised links between health and wellbeing [95], future research should explore these associations in order to identify any quantitative measurements of health that can be used as an indicator of wellbeing. By addressing these gaps, it may be possible to use low-cost IEQ sensors, wearable devices and cloud-based platforms to create holistic, personalised and scalable wellbeing monitoring systems.

## 8. Conclusions

Modern adaptive comfort models are beginning to recognise the value of the individual and to make environmental monitoring feasible outside of research; there is a need to explore low-cost, scalable solutions. Though, researchers should be aware that whilst that there are some accepted measurement factors for IEQ, there is no standardised definition that is universally accepted. Whilst it is important for researchers to understand the level of accuracy required by their study and procure sensors that provide this accuracy. It is also important, where possible, for researchers to consider the practical implications of their work and aim to procure equipment that can be pragmatically implemented by practitioners outside of a research setting. 

Rapid growth is driving down the cost and accessibility of IoT hardware and the software that supports it, many of these platforms are agnostic to hardware meaning they can be used as a holistic platforms that can collect, store and often analyse data from that are disparate and heterogeneous. These platforms also have the potential to collect data from wearables, which were discovered to be demonstrating values to environmental research within buildings. By virtue of enabling researchers and practitioners to measure individuals, wearables can enable and enhance the localisation of environmental studies within buildings and begin exploring holistic personalised approaches to health monitoring in buildings that analyse the individual as they move between environments.

## Figures and Tables

**Table 1:** List of Search Terms (Filters).

1	(Well?Being OR Wellbeing) **^‡^**
2	(IEQ OR “Indoor (Environment OR Environmental) Quality”)
3	(IAQ OR “Indoor Air Quality”)
4	(“Sick Building Syndrome” OR SBS)
5	“(Thermal OR Visual OR Acoustic) Comfort”
6	Indoor Pollution
7	(Arduino OR “Raspberry Pi” OR “rPi”)
8	Sensors
9	(“State?of?the?art” OR Industrial OR “Scientifically Valid*”) **^‡^**
10	(“Low Cost” OR DIY OR Cheap)
11	(Heating Ventilation Air Conditioning OR HVAC)
12	Wearable
13	(POE OR “Post?Occupancy Evaluation”) **^‡^**
14	Building
15	“Building Design”
16	“Green Building”
17	“Built Environment”
18	Office
19	Workplace
20	“Commercial Building”
21	Housing
22	Residential

**^‡^** The ‘single-character’ search wildcard ‘?’ was used on all databases except Google Scholar, which requires the ‘~’ wildcard instead.

**Table 2:** Overview of Measurements in Selected Studies.

	Ref	Year	Building Type	Duration	Sample Size ^†^	Demographics	Research Focus	IAQ ^‡^	VC ^§^	AC ^¶^	TC ^††^	SotA ^‡‡^	LCS ^§§^	DIY ^¶¶^	WS ^†††^	BMS ^‡‡‡^
1	Rogage et al. [35]	2019	Residential (Multi-unit)	6 months	-	Residents from 7 flats, multi-unit social home building	IEQ/OC/STP	-	-	-	√	√	-	√	-	-
2	Clements et al. [36]	2019	Commercial (Office)	18 weeks	8	Office workers	OC	√	√	√	√	√	-	√	√	√
3	Ghahramani et al. [37]	2019	Education (University)	1 day	41	18–24-Year-old students uniformly random mix gender	OP	√	-	-	-	√	-	-	√	-
4	Parkinson et al. [20]	2019	Commercial (Office)	3 months	-	-	IEQ	√	√	√	√	√	-	√	-	√
5	Coleman and Meggars [38]	2018	Education (University)	8 days	-	-	STP	√	-	-	√	-	-	√	-	-
6	Moreno-Rangel et al. [26]	2018	Residential (Flat)	4 days	-	-	STP	√	-	-	-	√	√	-	-	-
7	Tiele et al. [39]	2018	Laboratory	3 days	-	-	STP	√	√	√	√	-	-	√	-	-
8	Tijani et al. [40]	2018	Laboratory	1 day	-	-	STP	√	-	-	√	-	-	√	-	-
9	Broderick et al. [23]	2017	Residential (Single-Family)	1 day	55	Non-smoking family with one or two adults and children. The average occupancy of 3.7 per household	IEQ	√	-	-	√	√	-	√	-	-
10	Földváry et al. [28]	2017	Residential (Multi-Unit)	1 week, x2	94	One participant from each household	IEQ	√	-	-	√	√	-	-	-	-
11	Li et al. [32]	2017	Residential Commercial	6 weeks 3 weeks	37	--	OC	√	√	√	√	-	√	-	√	√
12	MacNaughton et al. [16]	2017	Commercial (Office)	5 days	109	Office workers aged 20-70 near equal male:female ratio	IEQ/OP	√	√	-	-	√	√	-	√	-
13	Tang et al. [10]	2017	Commercial (Office)	3 weeks	-	-	IEQ	√	-	-	√	-	-	√	-	-
14	Tanguy et al. [41]	2017	Residential (Single-Family)	-	8	-	STP	√	-	-	√	-	-	√	-	-
15	Tran et al. [42]	2017	Laboratory	-	-	-	STP	√	-	-	√	-	-	√	-	-
16	Ali et al. [43]	2016	Lab, Office, Outdoor	7 days	-	-	STP	√	-	-	√	√	-	√	-	√
17	Coombs et al. [21]	2016	Residential (Multi-Unit)	1 year	64	Predominantly African American 7–12-year-old asthmatic children from low-income families	IEQ	√	-	-	√	√	-	-	-	-
18	Allen et al. [34]	2016	Commercial (Office)	2 weeks/6 Days	30/24	Knowledge workers (professional grade employees)	IEQ/OP	√	√	-	-	√	√	-	-	-
19	Marques and Pitarma [6]	2016	Laboratory	-	-	-	STP	√	√	-	-	-	-	√	-	-
20	MiHai and Iordache [44]	2016	Education (University)	5 hours	115	Students and teachers	IEQ	√	√	√	√	√	-	-	-	-
21	Mui et al. [7]	2016	Commercial (Office)	-	-	-	IEQ	√	√	√	√	√	-	√	-	√
22	Shan et al. [45]	2016	Education (University)	2 days	39	University Students with 6:7 male-female ratio	IEQ/OP	√	-	-	√	√	-	-	-	-
23	Salamone et al. [46]	2015	Laboratory	3 days	-	-	STP	√	√	√	√	-	-	√	-	-
24	Hua et al. [27]	2014	Education (University)	4 weeks	46	20 - 50-year-old students and staff members, with the majority being between 20–29 years old	IEQ/OC	√	√	√	√	√	-	-	-	-
25	McGill et al. [22]	2014	Residential (Multi-Unit)	1 day, x2	13	3 properties with an average of four people per house and at least one smoker in the family - non-smoking	IEQ/OC	√	-	-	-	√	-	-	-	-
26	De Giuli et al. [4]	2012	Education (School)	1 day	-	Primary school children from seven Italian schools	IEQ/OC	√	√	√	√	√	-	-	-	-
27	Painter Brown et al. [24]	2010	Commercial (Office)	1 month	-	-	STP	√	-	-	√	√	-	-	-	-

^†^ Sample size refers to the number of people measured in each study. ^‡^ Indoor Air Quality. ^§^ Visual Comfort. ¶ Acoustic Comfort. †† Thermal Comfort. ‡‡ State-of-the-Art. §§ Low-Cost Sensors. ¶¶ ‘Do It Yourself’ Sensors (Standalone electronic sensing components, often run through Arduino/Raspberry Pi). ††† Wearable Sensors. ‡‡‡ Building Management System. **Research Focus Key:** OC: Occupant Comfort. OP: Occupant Performance. IEQ: Indoor Environment Quality. STP: Sensor Technology Performance.

**Table 3:** State-of-the-art sensors.

		IAQ ^†^					TC ^‡^			VC ^§^		AC ^¶^
Manufacturer	Model	CO_2_^††^	CO ^‡‡^	H_2_CO ^§§^	PM ^¶¶^	VOC ^†††^	Temp	Air Velocity	RH ^‡‡‡^	Lux	Light Colour	Sound
SKC	AirChek 2000 [21]	-	-	-	-	-	-	√	-	-	-	-
Bruel and Kjaer	1213 [4]	-	-	-	-	-	√	√	√	-	-	-
	2250 [44]	-	-	-	-	-	-	-	-	-	-	√
CO2Meters	CM-0018AA [45]	√	-	-	-	-	√	-	√	-	-	-
Extech	SD800 data logger [27]	√	-	-	-	-	√	-	√	-	-	-
	EA80 data logger [22]	√	-	-	-	-	√	-	√	-	-	-
Fieldpiece	SCM4 [20]	√	-	-	-	-	-	-	-	-	-	-
GrayWolf	FM-108 [23]	-	-	√	-	-	√	-	-	-	-	-
	IQ-410 [26]	√	√	-	-	√	√	-	√	-	-	-
	IQ-610 [23]	√	√	-	-	√	√	-	√	-	-	-
	PC-3016A [26]	-	-	-	√	-	√	-	√	-	-	-
	TG-502 [23,26]	-	-	-	-	√	√	-	√	-	-	-
HalTech	HFX205 [20]	-	-	√	-	-	√	-	√	-	-	-
HOBO	U12-012 [43]	-	-	-	-	-	√	-	√	√	-	-
Konica Minolta	CL-500A [36]	-	-	-	-	-	-	-	-	√	√	-
Lascar	EL-USB-CO [23]	√	-	-	-	-	-	-	-	-	-	-
Monnit Corp	Wireless Humidity Sensor [36]	-	-	-	-	-	-	-	√	-	-	-
	Wireless Temp Sensor [36]	-	-	-	-	-	-	-	-	-	-	-
NTi Audio	XL2 Analyzer [36]	-	-	-	-	-	-	-	-	-	-	√
Rion	NL-52 [20]	-	-	-	-	-	-	-	-	-	-	√
Telaire	7000 [43]	√	-	-	-	-	-	-	-	-	-	-
	7001 [7,23]	√	-	-	-	-	-	-	-	-	-	-
TSI	DustTrak II 8532 [20]	-	-	-	√	-	-	-	-	-	-	-
	Q-Trak 7575 [20,34]	√	√	-	-	√	√	-	√	-	-	-
	Q-Trak 964 [36]	-	-	-	-	-	√	√	√	-	-	-
	SidePak AM510 [23]	-	-	-	-	√	-	-	-	-	-	-
	Velocicalc 9545 [45]	-	-	-	-	-	√	√	√	-	-	-
Watson	N-8681 SOLAR [22]	-	-	-	-	-	√	√	√	√	-	-
Wilks	InfraRan Specific Vapor Analyzer [45]	√	√	√	-	-	-	-	-	-	-	-
Wholër	CO_2_ datalogger [22]	√	-	-	-	-	-	-	-	-	-	-
Wovyn	Lux1000	-	-	-	-	-	-	-	-	√	-	-
Wovyn	Color Lux1000	-	-	-	-	-	-	-	-	√	√	-

Table outlines state-of-the-art sensors used within reviewed studies, outlining the manufacturers, models and measurement, factors. These sensors cost range from several hundreds of pounds to several thousand. ^†^ Indoor Air Quality. ^‡^ Thermal Comfort. ^§^ Visual Comfort. ^¶^ Acoustic Comfort. ^††^ Carbon Dioxide. ^‡‡^ Carbon Monoxide. ^§§^ Formaldehyde. ^¶¶^ Particulate Matter (PM1.0/PM2.4/PM10). ^†††^ Volatile Organic Compounds. ^‡‡‡^ Relative Humidity.

**Table 4:** Low-cost sensors.

				IAQ ^†^					TC ^‡^		VC ^§^	AC ^¶^
Manufacturer		Sensor	Cost ^††^	CO_2_^‡‡^	eCO_2_^§§^	CO ^¶¶^	PM ^†††^	VOC ^‡‡‡^	Temp	RH ^§§§^	Lux	Sound
Adafruit		DHT22 [38,46]	£2–£5	-	-	-	-	-	√	√	-	-
		MAX 4466 [39]	£1–£7	-	-	-	-	-	-	-	-	√
Amphenol		T6615 [6]	£80	√	-	-	-	-	-	-	-	-
		T6713 [38,39]	£70–£75	√	-	-	-	√	-	-	-	-
AMS		CCS811 [39]	£6-£30	-	√	-	-	√	-	-	-	-
		iAQ-Core C [39]	£15–£30	-	√	-	-	√	-	-	-	-
		TSL2561 [39,43]	£4–£7	-	-	-	-	-	-	-	√	-
BuildAX		Wireless Building Monitoring System [47]	£90	-	-	-	-	-	√	√	√	-
CO2 Meters.com		K-30 [7,43,46]	Price by quotation	√	-	-	-	-	-	-	-	-
GSS		COZIR [32]	£155	√	-	-	-	-	-	-	-	-
Hanwei		MQ7 [6,40]	£2–£7	-	-	√	-	-	-	-	-	-
Honeywell		HIH-4030 [40]	£10–£40	-	-	-	-	-	√	√	-	-
		HPMA115S0 [39]	£35–£45	-	-	-	√	-	-	-	-	-
Netatmo		Weather Station [16,34]	£130	√	-	-	-	-	√	√	-	√
Seeed Technology		MH-Z16 [36]	£65–£100	√	-	-	-	-	-	-	-	-
		MH-Z19 [36]	£15	√	-	-	-	-	-	-	-	-
		AM2302 [7]	£3–£15	-	-	-	-	-	√	√	-	-
		101020030 [7]	£3–£10	-	-	-	-	-	-	-	√	-
		101020023 [7]	£4–£6	-	-	-	-	-	-	-	-	√
Sensirion		SHT10 [6]	£2–£7	-	-	-	-	-	√	√	-	-
		SHT15 [43]	£4–£25	-	-	-	-	-	√	√	-	-
		SHT31 [39]	£3–£15	-	-	-	-	-	√	√	-	-
Sensorist		Wireless Pro T/RH [32]	£140	-	-	-	-	-	√	√	-	-
SGX SensorTech		MiCS-VZ-89TE [39]	£20–£25	-	√	-	-	√	-	-	-	-
Sharp		GP2Y1010AU0F [40]	£10–£15	-	-	-	√	-	-	-	-	-
Telaire		T6615 [6]	£80	√	-	-	-	-	-	-	-	-
		T6713 [38]	£75	√	-	-	-	-	-	-	-	-
Texas Instruments		LM35 [40]	£1	-	-	-	-	-	√	-	-	-

Table outlines low-cost sensors used within the reviewed studies, outlining the manufacturers, models, measurement factors and typical costs. ^†^ Indoor Air Quality. ^‡^ Thermal Comfort. ^§^ Visual Comfort. ^¶^ Acoustic Comfort. ^††^ Costs are approximate and taken from Google Shopping Search Engine – prices vary according to manufacturer and retailer. ^‡‡^ Carbon Dioxide. ^§§^ Equivalent CO2 (eCO2) is the measure used to communicate the global warming potential of combined greenhouse gasses. ^¶¶^ Carbon Monoxide. ^†††^ Particulate Matter (PM1.0/PM2.4/PM10). ^‡‡‡^ Volatile Organic Compounds. ^§§§^ Relative Humidity.

## Supplementary Materials

The following are available online at https://www.mdpi.com/1660-4601/17/11/3995/s1, Table S1: Preferred Reporting Items for Systematic reviews and Meta-Analyses extension for Scoping Reviews (PRISMA-ScR) Checklist.
